# Quantitative Analysis of Ligand-EGFR Interactions: A Platform for Screening Targeting Molecules

**DOI:** 10.1371/journal.pone.0116610

**Published:** 2015-02-27

**Authors:** Wei-Ting Kuo, Wen-Chun Lin, Kai-Chun Chang, Jian-Yuan Huang, Ko-Chung Yen, In-Chi Young, Yu-Jun Sun, Feng-Huei Lin

**Affiliations:** 1 Institute of Biomedical Engineering, National Taiwan University, Taipei, Taiwan; 2 Graduate Institute of Clinical Dentistry, National Taiwan University, Taipei, Taiwan; 3 Institute of Biomedical Engineering and Nanomedicine, National Health Research Institutes, Miaoli, Taiwan; Seoul National University, KOREA, REPUBLIC OF

## Abstract

Epidermal growth factor receptor (EGFR) is often constitutively stimulated in many cancers owing to the binding of ligands such as epidermal growth factor (EGF). Therefore, it is necessary to investigate the interaction between EGFR and its targeting biomolecules. The main aim of this study was to estimate the binding affinity and adhesion force of two targeting molecules, anti-EGFR monoclonal antibody (mAb LA1) and the peptide GE11 (YHWYGYTPQNVI), with respect to EGFR and to compare these values with those obtained for the ligand, EGF. Surface plasmon resonance (SPR) was used to determine the equilibrium dissociation constant (K_D_) for evaluating the binding affinity. Atomic force microscopy (AFM) was performed to estimate the adhesion force. In the case of EGFR, the K_D_ of EGF, GE11, and mAb LA1 were 1.77 × 10^−7^, 4.59 × 10^−4^ and 2.07 × 10^−9^, respectively, indicating that the binding affinity of mAb LA1 to EGFR was higher than that of EGF, while the binding affinity of GE11 to EGFR was the lowest among the three molecules. The adhesion force between EGFR and mAb LA1 was 210.99 pN, which is higher than that observed for EGF (209.41 pN), while the adhesion force between GE11 and EGFR was the lowest (59.51 pN). These results suggest that mAb LA1 binds to EGFR with higher binding affinity than EGF and GE11. Moreover, the adhesion force between mAb LA1 and EGFR was greater than that observed for EGF and GE11. The SPR and AFM experiments confirmed the interaction between the receptor and targeting molecules. The results of this study might aid the screening of ligands for receptor targeting and drug delivery.

## Introduction

Epidermal growth factor receptor (EGFR) and its ligand, epidermal growth factor (EGF), are overexpressed in many malignancies, including cancers of the head and neck, breast, kidney, lung, colon, ovary, prostate, brain and spine, pancreas, and bladder [1]. EGFR is activated when a ligand such as EGF binds to its extracellular domain and induces conformational changes. These changes cause the EGFR to form homodimers or heterodimers with other receptors [2]. This leads to the activation of the tyrosine kinase domain of EGFR and the auto-phosphorylation of its C-terminal tyrosine residues. These events subsequently lead to the activation of the downstream signaling pathway [3]. Overexpression of EGFR is associated with several hallmarks of cancer, including inhibition of apoptosis, sustained angiogenesis, proliferation and survival, and tissue invasion and metastasis [4]. Several EGFR inhibitors that block ligand binding have been developed. These inhibitors have been shown to arrest cell growth and induce apoptosis in cancer cells [5]. In recent years, researchers have focused on identifying targeting biomolecules for more efficient drug delivery. Identifying the ligand-receptor interactions will aid in the design of optimal targeting molecules and drugs for cancer therapy.

Surface plasmon resonance (SPR) and atomic force microscopy (AFM) are powerful techniques used to analyze biomolecular interactions [6]. The SPR biosensor technology is used to measure reaction kinetics and to calculate the affinity constants of biomolecular interactions [7, 8]. In this method, the receptor is immobilized on the activated surface of a sensor chip, and a solution containing the ligand is then flowed over the surface of the chip. Binding of the interacting ligand to the surface-immobilized receptors alters the mass of the surface layer. The corresponding change in the refractive index and the shift of the resonant angle of reflected light is then detected. These changes can be monitored in real time by plotting the resonance signal as a function of time [9, 10]. The AFM is a high resolution scanning machine [11], and it is also a useful tool for the measurement of the adhesion forces between ligands and receptors [12, 13]. In this method, the AFM tip is coated with the ligand while the receptor is immobilized on the substrate. The tip is then brought in contact with the surface of substrate, enabling the formation of the ligand-receptor complex. Subsequently, the tip is retracted from the surface and the rupture force required for the dissociation of the ligand from the receptor is determined by estimating the extent of deflection of the cantilever, detected by a laser beam aimed at the cantilever and reflected onto a photodiode detector [14].

In this study, we determined the binding affinity and adhesion force of two targeting biomolecules—anti-EGFR monoclonal antibody (mAb LA1) and peptide GE11 (YHWYGYTPQNVI)—to EGFR, and compared these values with those obtained for EGF, which is the main *in vivo* competitor for the receptor during clinical application. If the binding affinity and adhesion force of the targeting molecules to EGFR are higher than that of EGF, they could compete with EGF for EGFR binding and block the subsequent activation of cellular signaling pathways. Therefore, the SPR and AFM techniques can be used to screen molecules to discover new EGFR-binding molecules for efficient drug delivery.

## Materials and Methods

### Materials and reagents

Sensor CM5 chips, HBS-EP buffer (10 mM Hepes, 150 mM NaCl, 3mM EDTA, and 0.005% Tween-20), N-Hydroxysuccinimide (NHS), 1-Ethyl-3-(3-dimethylaminopropyl) carbodiimide (EDC), and ethanolamine hydrochloride (EA) were obtained from Biacore Life Science (Uppsala, Sweden). EGFR was purchased from Sigma-Aldrich (Missouri, USA). EGF was purchased from PeproTech (New Jersey, USA). Anti-EGFR monoclonal antibody (mAb LA1) was purchased from Millipore (Massachusetts, USA). Peptide GE11 with sequence YHWYGYTPQNVI was custom synthesized by Millipore (Massachusetts, USA). AFM tips were purchased from Asylum Research (California, USA).

### Preparation of EGFR on Sensor CM5 chips

There are dextran matrix covered with carboxyl groups on CM5 chip. The chips were equilibrated with HBS-EP buffer, followed by addition of 0.1 M NHS and 0.4 M EDC mixture to activate dextran matrix to create succinimide esters. EGFR was passed over at a concentration of 10 μg/ml in 10 mM sodium acetate (pH 4.5). The esters reacted spontaneously with amino groups of EGFR. And EA was added to block the residual N-hydroxysuccinimide ester.

### Binding affinity analysis

The binding affinity of EGF, peptide GE11 and anti-EGFR antibody to EGFR was measured by using a Biacore X SPR system (Biacore Life Science, Uppsala, Sweden) at room temperature. The chip without EGFR on the other flow channel was used as control. PBS was used as the running buffer and 50 mM NaOH was used for regeneration of the chip surface. For each concentration of EGF, peptide GE11 and anti-EGFR antibody, the flow rate of association and dissociation was 10 μl/min and regeneration was 100 μl/min. The concentrations were 0.25, 0.5, 1, 2, 4 μM for EGF, 10, 20, 40, 80, 160 μM for peptide GE11, and 0.75, 1.5, 3 μM for anti-EGFR antibody. The equilibrium dissociation constant (K_D_) was obtained to evaluate the binding affinity by using the BIAEvaluation software (Biacore Life Science, Uppsala, Sweden).

### Modification of AFM tips

EGF, peptide GE11 and anti-EGFR antibody would be individually immobilized on the succinimide-modified silicon nitride cantilever tips and coating was performed only on the extreming end of the cantilever to avoid any influence on the cantilever spring constant. The process was briefly described as followings. The tips were immersed in the solution of EGF, peptide GE11 and anti-EGFR antibody with concentration of 1 mg/ml, respectively, for 24 hours at 4°C and then washed phosphate buffer solution (PBS). Finally, the residual succinimide ester was deactivated by EA for 30 minutes at 4°C. The modified tips were stored in PBS at 4°C for later use.

### Adhesion force measurements

The topography and microstructure of the chip was obtained by MFP-3D-BIO AFM (Asylum Research, California, USA) with tapping mode. The binding force between the test sample and EGFR was measured on AFM by contact mode in PBS at room temperature. The location of immobilized EGFR on the chip was identified to allow tip moving on for later measurement. Stress-strain curve (force-distance curve) was obtained by moving the surface-modified tip to the EGFR-immobilized location, holding it on for several seconds to allow binding to occur and then retracting. All the measurements were executed at the same loading rate. The spring constant of cantilever was determined in air (measured values from 0.09 to 0.18 N/m). Curves showing significant non-specific interactions as well as those showing a zero interaction were not analyzed.

## Results

### Immobilization of EGFR on Sensor CM5 chips

**Fig 1:**
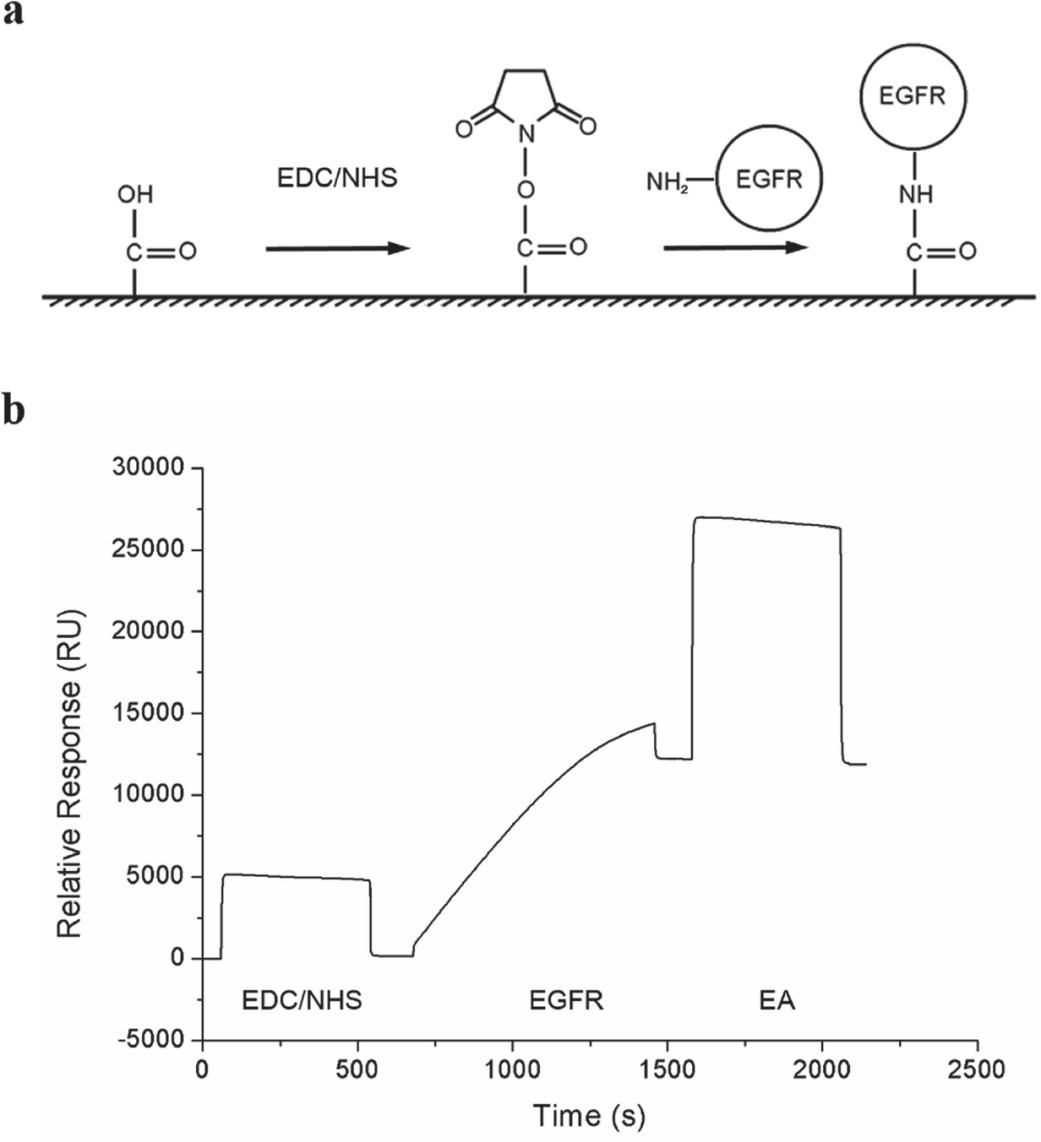
Immobilization of EGFR to the Sensor CM5 chips. (a) EGFR was immobilized to the surface of chip by amine coupling method. (b) SPR Sensorgram of the immobilization of EGFR.

EGFR was immobilized on Sensor CM5 chips using the amine coupling method (Fig. 1A). We used SPR to monitor the change in resonance units (RUs), i.e. change in the refractive index of the chip, at every second. First, ethyl(dimethylaminopropyl)carbodiimide/*N*-hydroxysuccinimide (EDC/NHS) were reacted with the carboxyl groups on the dextran layer of the chip. Then, EGFR was immobilized on the chip via the free primary amine groups. Finally, any residual free amine groups were blocked with ethanolamine (EA). Immobilization was considered complete when a value of 12,000 RU of EGFR was achieved on an experimental flow channel (Fig. 1B).

### Estimation of binding affinity by SPR

**Fig 2:**
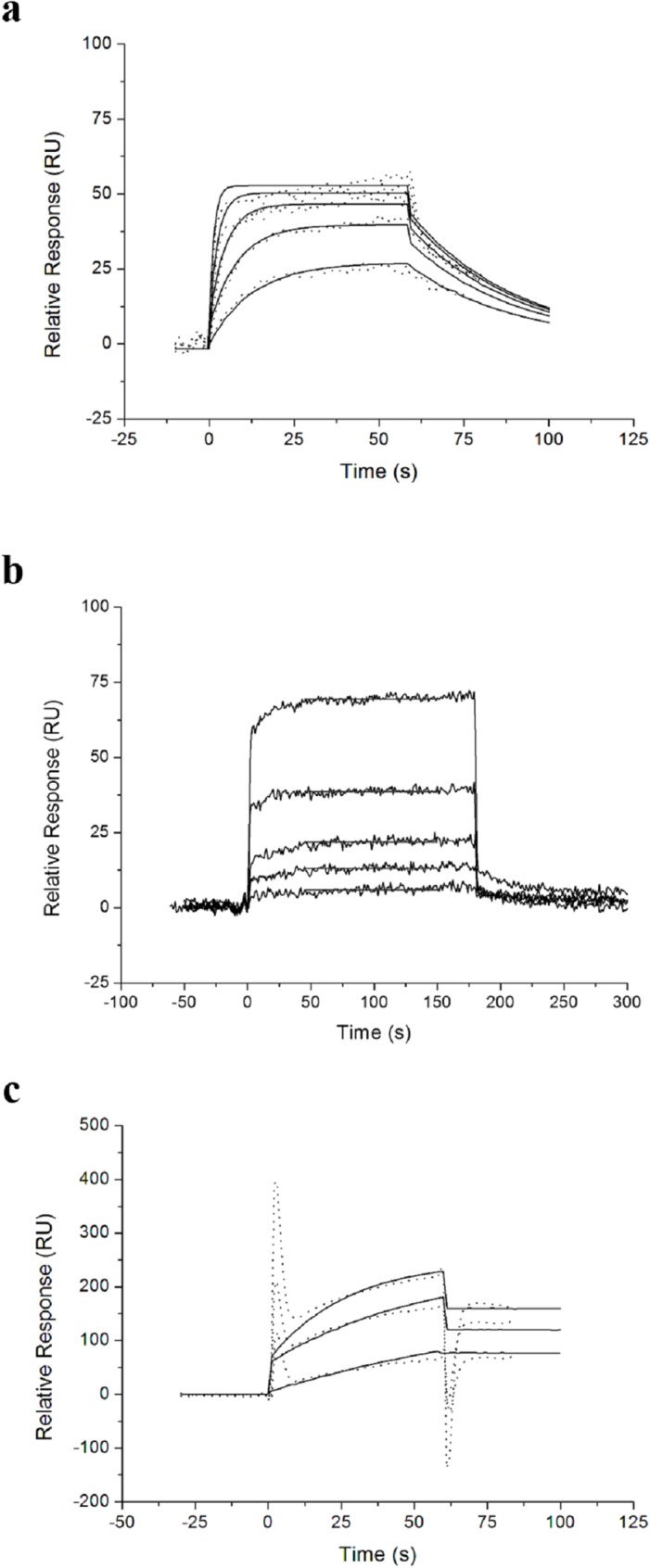
SPR sensorgrams of ligands to EGFR immobilized surface. (a) EGF to EGFR. (b) GE11 to EGFR. (c) mAb to EGFR.

The binding affinity between EGF and EGFR was estimated by flowing the ligand (EGF) over the EGFR-immobilized Sensor CM5 chip. Different concentrations of EGF were injected into the flow-channel and then passed over the EGFR-immobilized Sensor M5 chip. As shown in Fig. 2A, the binding affinity between EGF and EGFR was estimated in terms of the dissociation constant (K_D_), calculated using the BIA evaluation software (Biacore Life Science). The K_D_ value between EGF and EGFR was approximately 1.77 × 10^−7^ (Table 1).

**Table 1:** K_D_ of ligands to EGFR based on Fig. 2.

Ligands	EGF	GE11	mAb
K_D_ (M)	1.77 × 10^−7^	4.59 × 10^−4^	2.07 × 10^−9^

K_D_: equilibrium dissociation constant

The binding affinities of GE11 and mAb LA1 to EGFR were also estimated as described above (Fig. 2B and 2C). The K_D_ value between GE11 and EGFR was 4.59 × 10^−4^ and that between GE11 and EGFR was 2.07 × 10^−9^ (Table 1).

### Estimation of adhesion force by AFM

**Fig 3:**
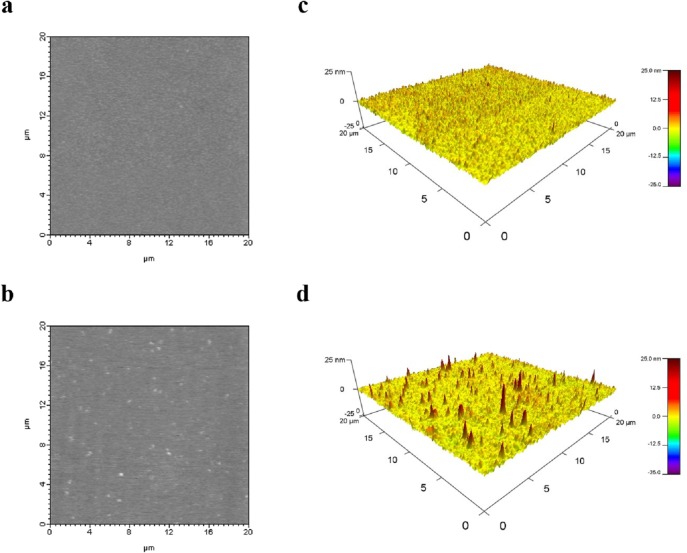
AFM pictures of Sensor CM5 chip surface and surface immobilized with EGFR. (a) The height image of chip. (b) The height image of EGFR immobilized chip. (c)(d) The three-dimensional view of (a) and (b) with a pseudocolor scale ranging from low (purple) to high (red).

**Fig 4:**
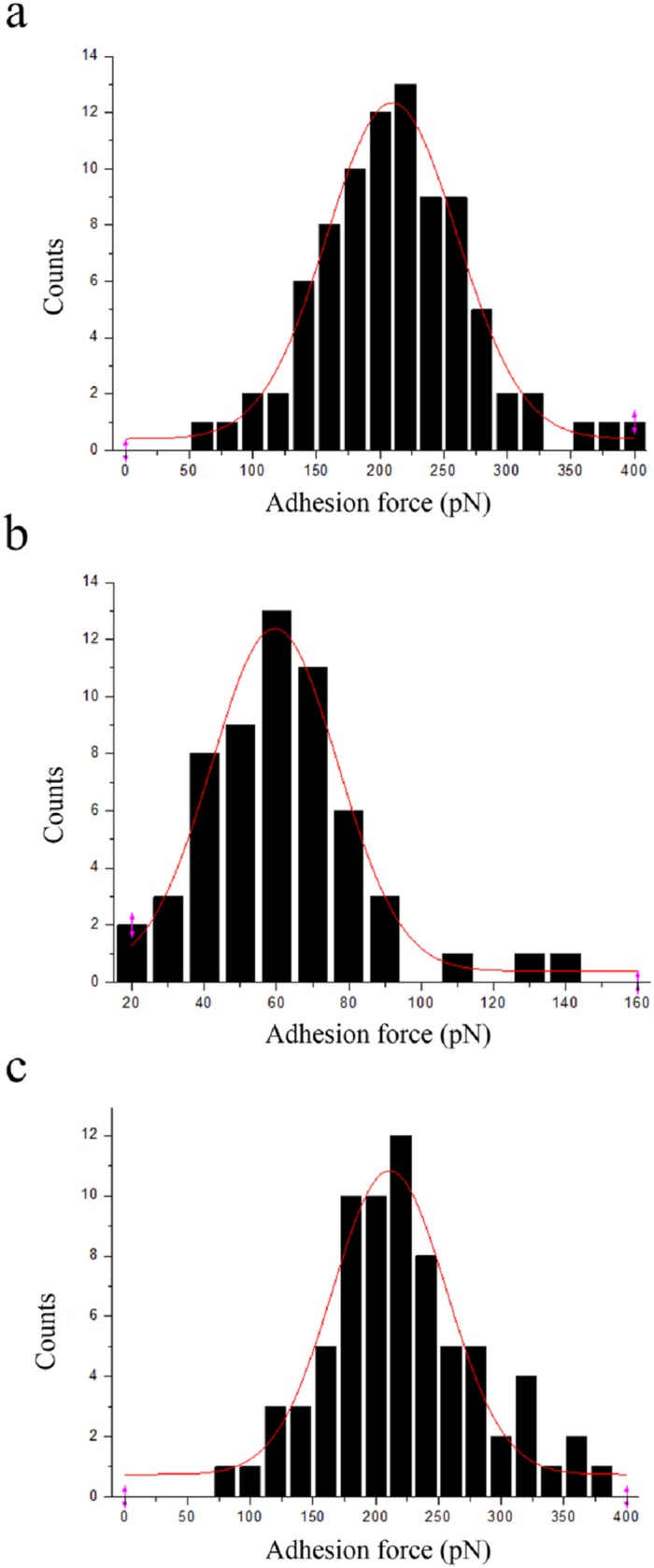
Adhesion force histograms with fitting Gaussian (red line) of ligands to EGFR immobilized surface. (a) EGF to EGFR. (b) GE11 to EGFR. (c) mAb to EGFR.

The tip of the AFM (coated with immobilized EGF) was mounted on the cantilever of the AFM and scanned on Sensor CM5 chips to trace the location of the immobilized EGFR. AFM images of the chip surface with and without immobilized EGFR are shown in Fig. 3. The chip without immobilized EGFR had a relatively smooth surface (Fig. 3A), while the chip coated with immobilized EGFR appeared rough (Fig. 3B). The AFM images were processed using the IGOR Pro MFP-3D software (Asylum Research). Clear pictures could be obtained after image processing for both the control (Fig. 3C) and EGFR-immobilized (Fig. 3D) chips. Spikes as high as 25 nm could be observed on the EGFR-immobilized chip (Fig. 3D), indicating the location of the EGFRs. The adhesion force between EGF and EGFR was calculated, sorted into a histogram, and fitted to a single Gaussian curve (Fig. 4A). The Gaussian peak of the histogram was located at 209.41 pN (Table 2).

**Table 2:** Adhesion force of ligands to EGFR based on Fig. 4.

Ligands	EGF	GE11	mAb
Adhesion force (pN)	209.41	59.51	210.99

The same procedure was used to calculate the adhesion force between the targeting molecules, GE11 and mAb LA1, and EGFR. As shown in Fig. 4B and 4C, the Gaussian peak of the histogram was located at 59.51 pN for GE11, and at 210.99 pN for mAb (Table 2).

## Discussion

Recently, increased understanding of the molecular mechanism of tumor biology has led to the development of EGFR-targeting biomolecules, which exhibit improved target selectivity toward cancer cells [15]. Nevertheless, not all EGFR targeting biomolecules are equally effective, although all of them were designed to target EGFR. EGFR is often constitutively stimulated in cancer cells owing to the binding of ligands such as EGF [16]. Therefore, it is necessary to investigate the interaction mechanism between EGFR and its targeting biomolecules, which are known bind to EGFRs with higher affinity than their ligands (EGF). In this study, we determined the binding affinity of two targeting molecules—mAb LA1 and GE11—to EGFR and determined the adhesion force between these molecules and the receptor. Moreover, we compared these values with those obtained for EGF.

We used the kinetic method to calculate the binding affinity by SPR [17–19]. This model illustrates the simplest mechanism of interaction between a ligand (A) and an immobilized receptor (B). The binding reaction of A and B can be represented by the following equation:

$$A+B\overset{k_{a}}{\underset{k_{d}}{↔}}AB$$ (1)

where *k*_*a*_ is the association rate constant and *k*_*d*_ is the dissociation rate constant.

The rate of product (*AB*) formation at time *t* is represented by the following equation:

$$\frac{d\left[AB\right]}{dt}=k_{a}\left[A\right]\left[B\right]-k_{d}\left[AB\right]$$ (2)

After reaction time *t* has elapsed, the equation may be represented as follows:

$$\left[B\right]={\left[B\right]}_{0}-\left[AB\right]$$ (3)

where [*B*]_0_ is the concentration of *B* at *t* = 0.

On combining Equations (2) and (3), the following equation is obtained:

$$\frac{d\left[AB\right]}{dt}=k_{a}\left[A\right]\left({\left[B\right]}_{0}-\left[AB\right]\right)-k_{d}\left[AB\right]$$ (4)

The number of *AB* complexes formed at the surface is proportional to the signal, *R*. Therefore, Equation (4) may be represented as follows:

$$\frac{dR}{dt}=k_{a}C\left(R_{\text{max}}-R\right)-k_{d}R$$ (5)

where *dR/dt* is the rate of formation of surface-associated complexes, *C* is the concentration of *A*, and *R*_*max*_ is the capacity of *A* bound to *B* at the surface. The integrated form of the rate equation may be represented as follows:

$$R_{t}=\frac{Ck_{a}R_{\text{max}}}{Ck_{a}+k_{d}}\left[1-e^{-\left\{\left(Ck_{a}+k_{d}\right)t\right\}}\right]$$ (6)

This integrated rate equation describes the association phase of the binding curve.

The rate of dissociation of the complex (*AB*) is represented by the following equation:

$$\frac{dR}{dt}=-k_{d}R$$ (7)

The integrated form of the rate equation is as follows:

$$R_{t}=R_{0}e^{-k_{d}t}$$ (8)

where *R*_*t*_ is the response at time *t* and *R*_*0*_ is the amplitude of the response.

We used Equations (6) and (8) to independently fit the data obtained for the association and dissociation phases, respectively. These equations predict the binding kinetic parameters. We flowed different concentrations of EGF, GE11, and mAb LA1 over the immobilized EGFRs, and then calculated the equilibrium dissociation constant (K_D_) using the BIAEvaluation software (Fig. 2). Our results revealed that the K_D_ of EGF-EGFR binding was higher than that of mAb-EGFR binding, and lower than that of GE11-EGFR binding (Table 1). There is an inverse relationship between K_D_ and affinity. Therefore, the binding affinity of mAb to EGFR was higher than that of EGF to EGFR. The binding affinity of GE11 to EGFR was the lowest among the three molecules.

Next, we used AFM to measure the adhesion force between the ligands and EGFR by recording a force curve, which is a plot of cantilever deflection (d), converted from a position-sensitive photo-diode (PSPD). This deflection distance, as a function of sample position along the z-axis, is then converted into the force (F) acting on the spring constant (k) of the cantilever tip, and can be represented by the Hooke’s law [20] as follows:

F=k×d (9)

The spring constant is determined from the individual frequency resonances and shape factors, and is expressed as follows:

$$k=2w\left(\pi fL\right)\sqrt{\frac{\rho^{3}}{E}}$$ (10)

where *w* is the width of the cantilever, *f* is the measured resonant frequency, *L* is the length of cantilever, *ρ* is the density of the cantilever material, and *E* is the elastic modulus (Young’s modulus) of the cantilever material [21]. In this force curve, the adhesion force is characterized as the maximum force required to facilitate the separation of the ligand-receptor partners after contact. Therefore, the adhesion force of the interaction between the ligand and receptor can be estimated by measuring the increase in the volume of the force [22]. In this study, we individually coated EGF, GE11, and mAb LA1 on the tip of the AFM and immobilized EGFR on a chip to determine the adhesion force between the ligand and the receptor (Fig. 3). The results revealed that the adhesion force of the mAb-EGFR interaction was higher than that of the EGF-EGFR interaction, while the adhesion force of the GE11-EGFR interaction was the lowest among the three molecules (Fig. 4; Table 2).

GE11 was synthesized by the random peptide phage display method as an EGFR-targeting ligand that could not activate the receptor [23]. It has been conjugated to many biomaterials that interact with EGFR efficiently and with high specificity for imaging purposes and for drug delivery to EGFR-overexpressing tumors [24–26]. Nevertheless, Abourbeh *et al*. demonstrated that the EGFR-binding affinity/inhibitory potency of EGF are several orders of magnitude higher than those of GE11 by *in vitro* radioactive binding studies [27]. This was in contradiction to the results obtained by Li *et al*., who first reported the synthesis the GE11 peptide [23]. In this study, we used two non-radioactive methods, SPR and AFM, to confirm that the binding affinity and adhesion force of GE11 to EGFR was lower than that of EGF to EGFR.

## Conclusions

The results of our study revealed that mAb LA1 had higher adhesion force and binding affinity to EGFR compared with EGF and GE11. SPR and AFM analyses confirmed the interaction between the receptor and the targeting ligands. The results of this study might aid in the screening of ligands for receptor targeting and drug delivery.
