# Correction: Quantitative Analysis of Ligand-EGFR Interactions: A Platform for Screening Targeting Molecules

**DOI:** 10.1371/journal.pone.0124981

**Published:** 2015-04-13

**Authors:** Wei-Ting Kuo, Wen-Chun Lin, Kai-Chun Chang, Jian-Yuan Huang, Ko-Chung Yen, In-Chi Young, Yu-Jun Sun, Feng-Huei Lin

The following information is missing from the Funding section: We thank the Ministry of Science and Technology for financially supporting this research under project NSC102-2221-E-002-036-MY3.
